# Prevention of Cell Death by Activation of Hydroxycarboxylic Acid Receptor 1 (GPR81) in Retinal Explants

**DOI:** 10.3390/cells11132098

**Published:** 2022-07-02

**Authors:** Rupali Vohra, Berta Sanz-Morello, Anna Luna Mølgaard Tams, Zaynab Ahmad Mouhammad, Kristine Karla Freude, Jens Hannibal, Blanca Irene Aldana, Linda Hildegaard Bergersen, Miriam Kolko

**Affiliations:** 1 Department of Drug Design and Pharmacology, University of Copenhagen, 2100 Copenhagen, Denmark; berta.morello@sund.ku.dk (B.S.-M.); anna_tams@hotmail.com (A.L.M.T.); zaynab.mouhammad@gmail.com (Z.A.M.); blanca.aldana@sund.ku.dk (B.I.A.); 2 Department of Veterinary and Animal Sciences, University of Copenhagen, 1870 Frederiksberg, Denmark; kkf@sund.ku.dk; 3 Department of Ophthalmology, Copenhagen University Hospital, Rigshospitalet, 2600 Glostrup, Denmark; 4 Department of Clinical Biochemistry, Bispebjerg Hospital, University of Copenhagen, 2400 Copenhagen, Denmark; jens.hannibal@regionh.dk; 5 Brain Energy Muscle Group, University of Oslo, NO-0318 Oslo, Norway; l.h.bergersen@odont.uio.no; 6 Center for Healthy Aging, University of Copenhagen, 2200 Copenhagen, Denmark

**Keywords:** GPR81, HCA1, retina, retinal ganglion cells, cell death, lactate, glucose, energy metabolism, glutamate excitotoxicity, ATP

## Abstract

Background: Progressive retinal ganglion cell (RGC) dysfunction and death are common characteristics of retinal neurodegenerative diseases. Recently, hydroxycarboxylic acid receptor 1 (HCA_1_R, GPR81) was identified as a key modulator of mitochondrial function and cell survival. Thus, we aimed to test whether activation of HCA_1_R with 3,5-Dihydroxybenzoic acid (DHBA) also promotes RGC survival and improves energy metabolism in mouse retinas. Methods: Retinal explants were treated with 5 mM of the HCA_1_R agonist, 3,5-DHBA, for 2, 4, 24, and 72 h. Additionally, explants were also treated with 15 mM of L-glutamate to induce toxicity. Tissue survival was assessed through lactate dehydrogenase (LDH) viability assays. RGC survival was measured through immunohistochemical (IHC) staining. Total ATP levels were quantified through bioluminescence assays. Energy metabolism was investigated through stable isotope labeling and gas chromatography-mass spectrometry (GC-MS). Lactate and nitric oxide levels were measured through colorimetric assays. Results: HCA_1_R activation with 3,5-DHBAincreased retinal explant survival. During glutamate-induced death, 3,5-DHBA treatment also increased survival. IHC analysis revealed that 3,5-DHBA treatment promoted RGC survival in retinal wholemounts. 3,5-DHBA treatment also enhanced ATP levels in retinal explants, whereas lactate levels decreased. No effects on glucose metabolism were observed, but small changes in lactate metabolism were found. Nitric oxide levels remained unaltered in response to 3,5-DHBA treatment. Conclusion: The present study reveals that activation of HCA_1_R with 3,5-DHBA treatment has a neuroprotective effect specifically on RGCs and on glutamate-induced retinal degeneration. Hence, HCA_1_R agonist administration may be a potential new strategy for rescuing RGCs, ultimately preventing visual disability.

## 1. Introduction

The lactate receptor, hydroxycarboxylic acid receptor 1 (HCA_1_R), also known as GPR81, was discovered in the brain in the early 2000s [1,2]. HCA_1_R activates the Gi-mediated pathway, thereby inhibiting adenylate cyclase followed by a decrease of the secondary messenger cyclic AMP (cAMP), thereby finally altering numerous intracellular mechanisms [2,3]. In this context, HCA_1_R activation has been linked with anti-inflammatory responses [4,5], protection against glutamate excitotoxicity [6,7], synaptic plasticity [8,9], memory formation [10], neuronal growth [11,12], and reduced calcium spikes in specific models of epilepsy [13].

Because of these beneficial effects, HCA_1_R has gained attention as a promising target for inducing neuroprotection. In particular, retinal neuroprotection is of great need, as several inner retinal conditions e.g., glaucoma and other optic neuropathies, are characterized by an incurable progressive loss of the innermost retinal neurons, the retinal ganglion cells (RGCs) [14,15,16,17]. The eye and especially the retina is a complex organ consisting of various cell layers, which contribute to the conversion of light into neuronal signals. Neuronal inputs are then transmitted through the unmyelinated RGC axons to the scleral channel and then entering the visual cortex for image interpretation. This process is highly energy demanding and requires a well-regulated energy metabolism [18]. Aerobic glycolysis is the process of glucose breakdown to pyruvate and/or lactate and two ATP molecules that account for 80–96% of total glucose consumption in the retina [19,20]. Both lactate and pyruvate are known retinal energy sources [21,22,23,24,25], which can be further metabolized in the vast number of retinal mitochondria [26,27], where most ATP is produced through oxidative phosphorylation after the tricarboxylic (TCA) cycle.

In addition, lactate is the endogenous ligand for HCA_1_R, and while plasma levels of lactate in other tissues of the body are too low to activate HCA_1_R [2] (EC_50_: 1–5 mmol/L [28]), the retinal levels of lactate reach between 5–50 mmol/L [29,30,31], providing sufficient activation of HCA_1_R. Lactate treatment of retinal cells in culture has also been shown to exhibit various protective effects, resulting in improved Müller glia function measured through kinetic uptake of glutamate and increased survival of both cultured Müller glia and RGCs in healthy and diseased experimental setups in the presence and absence of glucose, respectively [21,23]. Moreover, cultured Müller glia have also revealed increased glucose metabolism and mitochondrial function in response to HCA_1_R activation with 3,5- dihydroxybenzoic acid (DHBA) [22]. Similarly, HCA_1_R-silenced BxPC3 cells displayed reduced mitochondrial activity and cell survival [32].

Activation of HCA_1_R has recently also been shown to promote RGC axonal growth [33,34], suggesting that HCA_1_R may play an important role in development and preservation of the visual nervous system. However, the specific processes involved are yet to be elucidated.

HCA_1_R is expressed in the retina and more specifically on RGCs and Müller glia [33]. Thus, it is tempting to suggest that lactate-mediated retinal protection may occur through HCA_1_R activation in addition to the metabolism of lactate and its contribution as an energy source. In this study, we therefore aimed to investigate the link between HCA_1_R activation and RGC as well as retinal tissue survival and metabolism by treating retinal explants and/or wholemounts with the exogenous ligand, 3,5-DHBA, a metabolite of alkylresorcinols (α-resorcylic acid), which behaves as a HCA_1_R agonist [35].

## 2. Materials and Methods

### 2.1. Retinal Tissue Preparation and Treatment Conditions

C57/BL6J female mice, aged 8–12 weeks (Janvier, Le Genest-Saint Isle, France), were euthanized by cervical dislocation. Eyes were immediately removed and transferred to pre-heated 37 °C dissection media, Hank’s Balanced Salt Solution (HBSS) supplemented with 1% penicillin-streptomycin (10.000 U/mL). Retinas were carefully dissected from the anterior part of the eyes and from the scleras under a light microscope (Leica S4E). Directly after dissection, the whole retinas were used for immunohistochemistry (IHC). Alternatively, the retinas were divided into four equal pieces (retinal explants) and placed in transparent cell culture inserts for 6-well plates (pore diameter: 0.4 μm) with the RGC layer facing upwards for subsequent investigation of tissue viability, ATP quantification, lactate release concentrations, gas-chromatography mass-spectrometry (GC-MS), and nitric oxide measurements. Culture media [Neurobasal (Gibco Life Technologies, Roskilde, Denmark) containing 2% B-27 supplement (Fisher Scientific, Roskilde, Denmark), 1% N-2 supplement (Fisher Scientific, Denmark), 1% penicillin–streptomycin (Gibco, 10,000 U/mL), and 0.1% amphotericin B (Gibco, 250 μg amphotericin B/mL and 205 μg of sodium deoxycholate/mL)] as well as 6 mM glucose were added to the wells and the plates were kept in an incubator containing 5% CO_2_/95% O_2_ at 37 °C. Each retina was considered a biological sample. When dividing the retina into four equal pieces (A,B,C,D) originating from the same biological retina sample, each sample letter was incubated for a specific period of time (i.e., 2, 4, 24, or 72 h) along with additional quarters of retinas from other biological sample retinas in each incubation time point. In GC-MS experiments, the retinal explants were incubated for 2 h. In experiments with whole retinas (IHC staining), one whole retina was used for treatment and the other retina for control. All experiments were performed in the presence and absence of 5 mM of 3,5-DHBA, a HCA_1_R agonist-like treatment.

### 2.2. Determination of Tissue Viability in Retinal Explants

Retinal tissue survival was determined by the lactate dehydrogenase (LDH) cytotoxicity detection kit (Takara Bio; Saint-Germain-en-Laye, France) following the manufacturer’s instructions. Briefly, retinal explants were incubated with pre-heated culture media [Neurobasal (Gibco, Denmark) containing 2% B-27 supplement (Fisher Scientific, Denmark), 1% N-2 supplement (Fisher Scientific, Denmark), 1% penicillin–streptomycin (Gibco, 10,000 U/mL), and 0.1% amphotericin B (Gibco, 250 μg amphotericin B/mL and 205 μg of sodium deoxycholate/mL; Denmark)] as well as 6 mM glucose for 2, 4, 24, and 72 h in a humidified incubator with 5% CO_2_/95% O_2_ at 37 °C. Once the incubations were finalized, culture media were collected and LDH release was determined by absorbance readings at 490 nm on a microplate reader (Spectramax i3×, Molecular Devices; San Jose, CA, USA). LDH levels in the supernatant obtained after sonication and centrifugation (20.000 g for 20 min at 4 °C) of retinal explants were used to normalize LDH levels present in the culture media. Glutamate (15 mM) was added to an additional set of explants for 2 h and retinal tissue survival was determined as mentioned above.

### 2.3. Immunohistochemistry and Image Acquisition and Analysis

After isolation of the retina from the anterior segment and the sclera, the corpus vitreum was stained with trypan blue and carefully removed. Whole-mounted retinas were then treated with 5 mM of 3,5-DHBA compared to an untreated control and rinsed with PBS followed by fixation with 4% paraformaldehyde for 8–12 h at 4 °C. Retinas were then permeabilized with 0.1% Triton X-100 in PBS for 1 h at room temperature (RT) and blocked with 2% bovine serum albumin (BSA, Sigma-Aldrich; Denmark) in HBSS (Gibco) for 1 h at RT. Subsequently, retinas were incubated for 48 h at 4 °C with the primary antibody rabbit anti-RNA-binding protein with multiple splicing [RBPMS; Novus Biologicals, catalog (cat.) number: NBP2-20112; Centennial, CO, USA] diluted 1:500 in PBS. After several washes with PBS, retinas were incubated overnight at 4 °C with the secondary antibody Alexa Fluor 488 goat anti-rabbit (Invitrogen, cat. number: A-11034; Waltham, MA, USA) diluted 1:500 in 2% BSA in HBSS (Gibco). Finally, retinas were mounted on glass slides with mounting medium (49.95% glycerol: 49.95% PBS: 0.1% 4′,6-diamidino-2-phenylindole (DAPI; Invitrogen, cat. number: D3571; Waltham, MA, USA), *v*:*v*:*v*). After drying out at RT protected from light, glass slides were stored at 4 °C until imaging.

Images were obtained with an iMIC confocal microscope (Till Photonics; Riga, Latvia) equipped with an Andromeda spinning disk system (Till Photonics; Riga, Latvia) and a Hamamatsu 16-bit camera (Model C10600-10B-H, Hamamatsu Photonic; Hamamatsu city, Japan). Retinal ganglion cell (RGC) quantifications were performed using FIJI Image J [36]. RBPMS-positive cells (RGCs) were counted manually in the superior, inferior, and lateral fractions of the retina, in both the central region of the retina surrounding the optic nerve head as well as in the periphery (Figure 2a). Central and peripheral zones were defined as 0–900 μm and 1700–2400 μm from the optic nerve head (ONH), respectively (Figure 2a). Within each retinal area (e.g., superior central), the number of RGCs in two regions of interest (ROI) was counted with acceptance of a maximum 10% difference between the two areas. If the difference between the two selected areas was above 10%, then a recount in two new selected areas was carried out. The average value of the two ROI per area per retina was then calculated.

### 2.4. Quantification of Intracellular ATP

Retinal explants were incubated for 2, 4, 24, and 72 h as described in the section “Retinal Tissue Preparation and treatment conditions”. Retinal intracellular ATP concentrations were measured using the Bioluminescent Somatic Cell Assay Kit (cat no. FLASC-1KT, Sigma-Aldrich; Denmark) following the manufacturer’s protocol. The bioluminescent signal proportional to the ATP present in each biological sample was measured at 560 nm in a microplate reader (Spectramax i3x, Molecular Devices). Intracellular ATP levels were normalized to protein concentration, which was calculated by the BCA protein assay (Sigma-Aldrich; Denmark).

### 2.5. Quantification of Extra Retinal Lactate Concentrations

Retinal explants were incubated for 2, 4, 24, and 72 h as described in the “Retinal Tissue Preparation and treatment conditions”. After incubation, the media was transferred to a 10 KDa spin filter (Amicon Ultra4, Merck Milipore, cat no.: UFC801024, Søborg, Denmark) for 15 min at 4000 *g* at 4 °C to remove lactate dehydrogenase. Extra retinal lactate concentrations were hereafter measured using colorimetric detection through the Lactate Assay Kit (cat. no. MAK064-1KT, Sigma-Aldrich; Denmark) according to the manufacturer’s protocol. Absorbance was read at 560 nm. Extra retinal lactate levels were normalized to protein concentration, which was calculated by the BCA protein assay (Sigma-Aldrich; Denmark).

### 2.6. Retinal Explant Incubations with [U-^13^C]Glucose or [U-^13^C]Lactate and Analysis by Gas Chromatography-Mass Spectrometry

Retinal explants were pre-incubated for 30 min with pre-heated glucose-free culture media [Neurobasal (Gibco) containing 2% B-27 supplement (Fisher Scientific, 50×), 1% N-2 supplement (Fisher Scientific, 100×), 1% penicillin–streptomycin (Gibco, 10,000 U/mL), and 0.1% amphotericin B (Gibco, 250 μg amphotericin B/mL and 205 μg of sodium deoxycholate/mL)] in a humidified incubator with 5% CO_2_/95% O_2_ at 37 °C. Retinal explants were then washed once with warm phosphate-buffered saline (PBS) and incubated for 2 h at 37 °C with cultured media containing 6 mM [U-^13^C]glucose (Cambridge Isotopes Laboratories Inc.; cat. no. CLM-1396-10) or 10 mM [U-^13^C]lactate (Cambridge Isotopes Laboratories Inc.; cat. no. CLM-1579-PK) with and without the addition of 5 mM of 3,5 DHBA.

Incubations were terminated by rinsing the retinas twice with ice-cold PBS. Subsequently, retinas were placed in ice-cold 70% ethanol, sonicated, and centrifuged (20,000 *g* for 20 min at 4 °C) to separate the soluble extract (supernatant) from insoluble components (pellet). The supernatants were lyophilized and stored at −20 °C until further spectrometric analysis by GC-MS. The protein concentration of pellets was determined by the BCA protein assay (Sigma-Aldrich).

Metabolic mapping of [U-^13^C]glucose and [U-^13^C]lactate in the retina using GC-MS was performed as previously described [21,22,23,37,38]. Briefly, retinal extracts were reconstituted in water, acidified to pH 1–2 with HCl, and evaporated to dryness under nitrogen flow. Analytes were extracted into an organic phase using 96% ethanol and benzene and derivatized using 14% dimethylformamide (DMF) and 86% *N*-(t-butyldimethylsilyl)-*N*-methyltrifluoroacetamide (MTBSTFA) with a modified procedure from Mawhinney et al. [39]. Standards containing unlabeled metabolites of interest and tissue extracts were separated and analyzed in a gas chromatograph (Agilent Technologies 7820A chromatograph, J&W GC column HP-5MS, parts no. 19091S-433; Santa Clara, CA, USA) coupled to a mass spectrometer (Agilent Technologies, 5977E; Santa Clara, CA, USA). The isotopic enrichment of the metabolites of interest was corrected for the natural abundance of ^13^C using the unlabeled standards and calculated according to *Biemann* [40]. Data are presented as labeling (%) of *M*+*X*, where *M* is the mass of the unlabeled molecule and *X* is the number of ^13^C-labeled carbon atoms in each metabolite.

### 2.7. Quantification of Extra Retinal Nitric Oxide Concentrations

Retinal explants were incubated for 2, 4, 24, and 72 h as described in the “Retinal Tissue Preparation and treatment conditions”. After incubation, the media was transferred to a 10 KDa spin filter (Amicon Ultra4, Merck Milipore, cat no.: UFC801024) for 15 min at 4000 *g* at 4 °C. Extra retinal nitric oxide concentrations were hereafter measured using colorimetric detection through the nitric oxide assay (cat. no. ADI-917-020, Enzo Life Sciences; Farmingdale, NY, USA) according to the manufacturer’s protocol. Absorbance was read at 550 nm. Extra retinal nitric oxide levels were normalized to protein concentration, which was calculated by the BCA protein assay (Sigma-Aldrich; Denmark).

### 2.8. Statistics

Statistical data analysis was performed using GraphPad Prism software (GraphPad Prism version 9.0; San Diego, CA, USA). The normality of data distribution was analyzed by Shapiro–Wilk’s test. When comparing two conditions, normally distributed data were analyzed by two-tailed Student’s *t*-test, whereas non-normally distributed data were analyzed by two-tailed Mann–Whitney test. More than two conditions were compared using one-way ANOVA followed by either Tukey’s multiple comparison post-test or Dunnett’s multiple comparison post-test to evaluate statistical differences between the experimental groups or compared to a common control, respectively. When comparing two or more parameters between two or more conditions, a two-way ANOVA with Sidak or Tukey’s correction was carried out. Outliers were detected using Grubb’s outlier test and subsequently removed from further analyses. In all analyses, *p* < 0.05 is considered statistically significant.

## 3. Results

### 3.1. HCA_1_R Activation Increases Retinal Explant Viability and Protects Retinas against Glutamate Excitotoxicity

**Figure 1:**
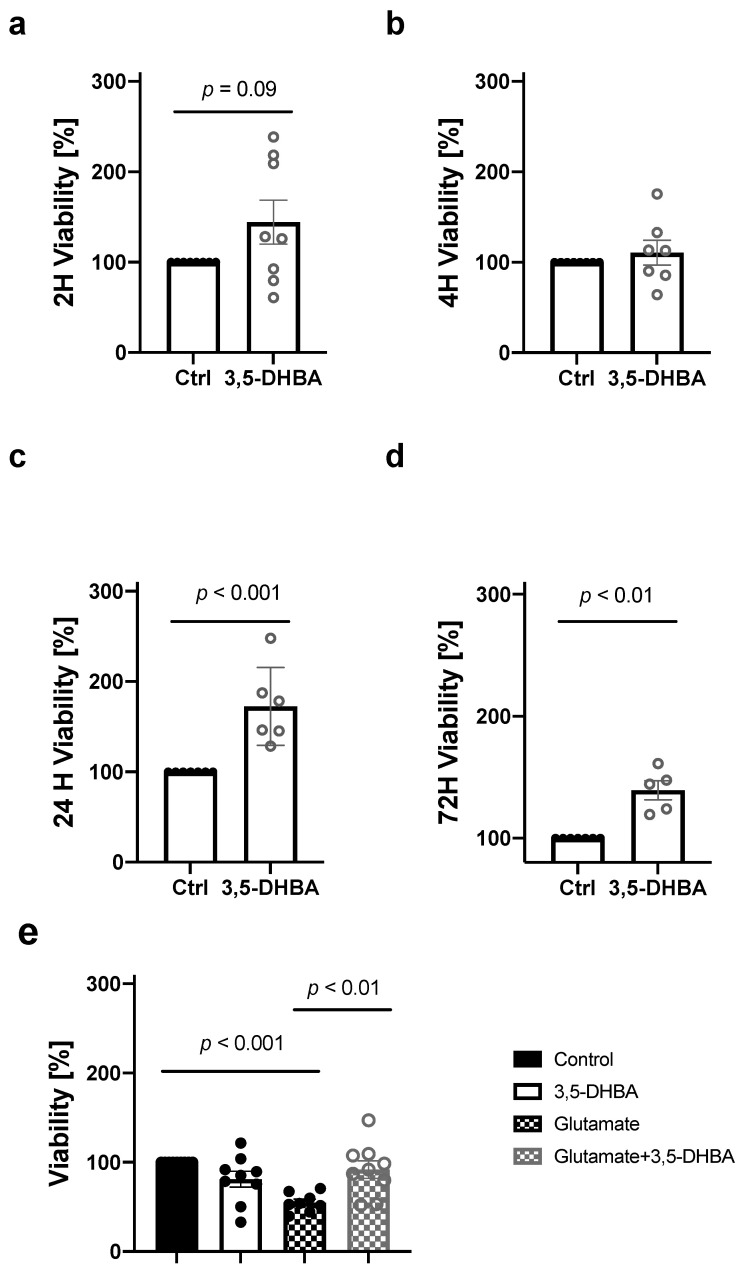
Retinal tissue viability is increased in response to 3,5-DHBA treatment. (**a**–**d**) Cell survival measured as lactate dehydrogenase (LDH) release from retinal explants incubated for 2, 4, 24, and 72 h. (**e**) Cell survival decreased during l-glutamate exposure, whereas the addition of 3,5-DHBA to glutamate-incubated retinas resulted in increased survival. Values are presented as mean ± S.E.M (*n* = 5–8 retinal quarters originating from five to eight different whole retinas). Statistics: Unpaired *t*-test for figure a–d. One-way ANOVA followed by Tukey’s multiple comparisons test for Figure 1e.

HCA_1_R has been linked to promoting cellular survival in numerous cultured cells [32,41]. To investigate the impact of HCA_1_R activation on retinal tissue viability, we incubated retinal explants with 5 mM of 3,5-DHBA, for 2, 4, 24, and 72 h followed by lactate dehydrogenase (LDH) measurements associated with cell membrane damage and cell death. We observed a tendency towards increase in retinal tissue viability in response to 3,5-DHBA treatment after 2 h, and a significant increase after 24 and 72 h (Figure 1a,c,d), whereas no significant change in retinal viability was observed at 4 h (Figure 1b). At 2 h, we also induced an insult to the retina by adding 15 mM of l-glutamate to mimic conditions of glutamate excitotoxicity that has previously been associated with retinal cell death [42,43,44]. Glutamate exposure decreased retinal viability. However, the addition of the 3,5-DHBA to glutamate-exposed retinas resulted in increased viability (Figure 1e). These results indicate that retinal protection and preservation occur in response to 3,5-DHBA treatment and that activation of HCA_1_R protects the retina from glutamate excitotoxicity.

### 3.2. Retinal Ganglion Cell (RGC) Survival Increases in Response to HCA_1_R Activation

**Figure 2:**
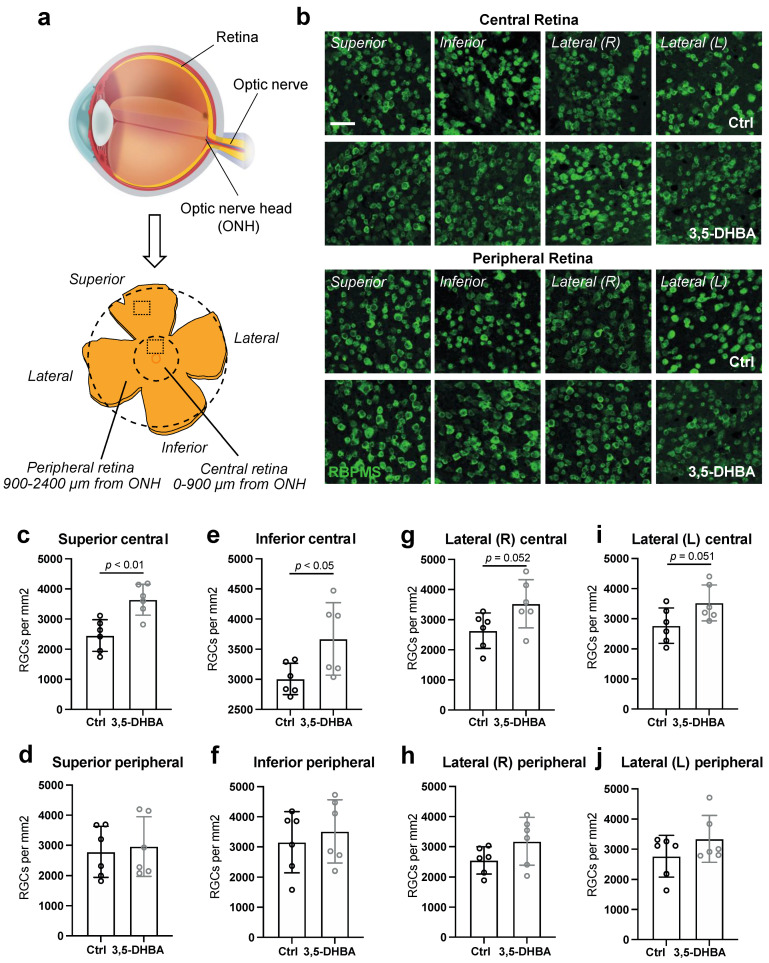
Retinal ganglion cell (RGC) quantity is increased in central parts of the retina in response to 3,5-DHBA treatment. (**a**) Graphical illustration of the cross-sectional eye and a retinal wholemount presenting the areas in which RGC quantification was performed (Modified from Tams et al. [46]). (**b**) Representative microscopy images of RBPMS staining of RGCs in the central and peripheral superior, inferior, and lateral regions. (**c**–**j**) RGC quantification (RGCs/mm^2^). Amount of RGCs is elevated in central parts of the retina in response to 3,5-DHBA treatment and remain unchanged in the peripheral subregions. Values are presented as mean ± S.D (*n* = 6 regions of interest (ROI), where two ROI come from the same retina, and a total of three retinas from three different mice was used for both the treatment and control). Statistics: Unpaired *t*-test. Scale bar: 100 μm. ONH: optic nerve head.

RGCs are the innermost cells of the retina and their axons comprise the optic nerve, by which visual nerve signals are transduced to the visual centers of the brain for interpretation [45]. To investigate whether an overall increase in retinal tissue survival corresponded to an increase in RGC survival, we quantified RBPMS-positive neurons, RGCs, in well-defined regions of the retinal wholemounts treated with and without 5 mM of 3,5-DHBA. We revealed a significant elevation in the number of RGCs in the superior and inferior regions of the central retina in response to 3,5-DHBA treatment (Figure 2c,d). A tendency towards increased amounts of RGCs was also seen in the right and left lateral part of the central regions (Figure 2e,f). No significant changes were seen in the peripheral retinal regions (Figure 2g–j). Thus, neuroprotection of RGCs is induced in the central retinal regions in response to 3,5-DHBA treatment.

### 3.3. Retinal ATP Levels Increase in Response to HCA_1_R Activation

**Figure 3:**
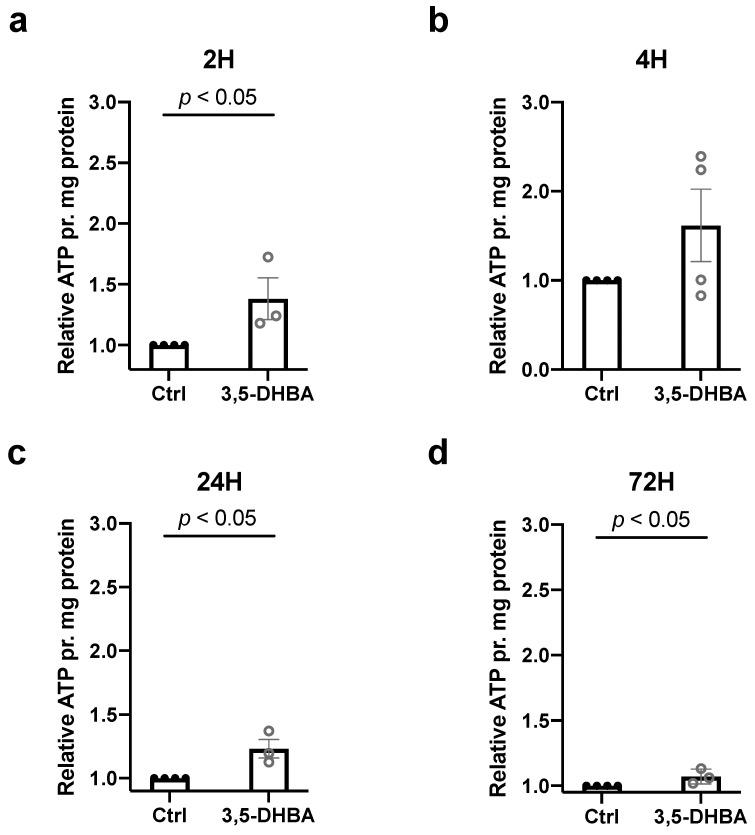
ATP levels in retinal explants are increased in response to 3,5-DHBA treatment. (**a**–**d**) Intracellular content of ATP measured in retinal explants incubated for 2, 4, 24, and 72 h via the Bioluminescent Somatic Cell Assay Kit. ATP levels increase during 2, 24, and 72 h of incubation with 3,5-DHBA compared to controls. Values are normalized to mg of protein in each sample. Values are presented as mean ± S.E.M (*n* = 3–4 retinal quarters originating from three to four different whole retinas). Statistics: Unpaired *t*-test.

To assess whether an increase in retinal survival corresponded with elevated energy production, we measured intracellular ATP contents in retinal explants incubated for 2, 4, 24, and 72 h with 3,5-DHBA. We observed an increase in ATP levels at all timepoints except for 4 h of incubation (Figure 3), demonstrating that ATP levels increase in response to 3,5-DHBA treatment. Higher ATP levels may therefore underlie increased retinal tissue viability.

### 3.4. Extra-Retinal Lactate Levels Decrease in Response to HCA_1_R Activation

**Figure 4:**
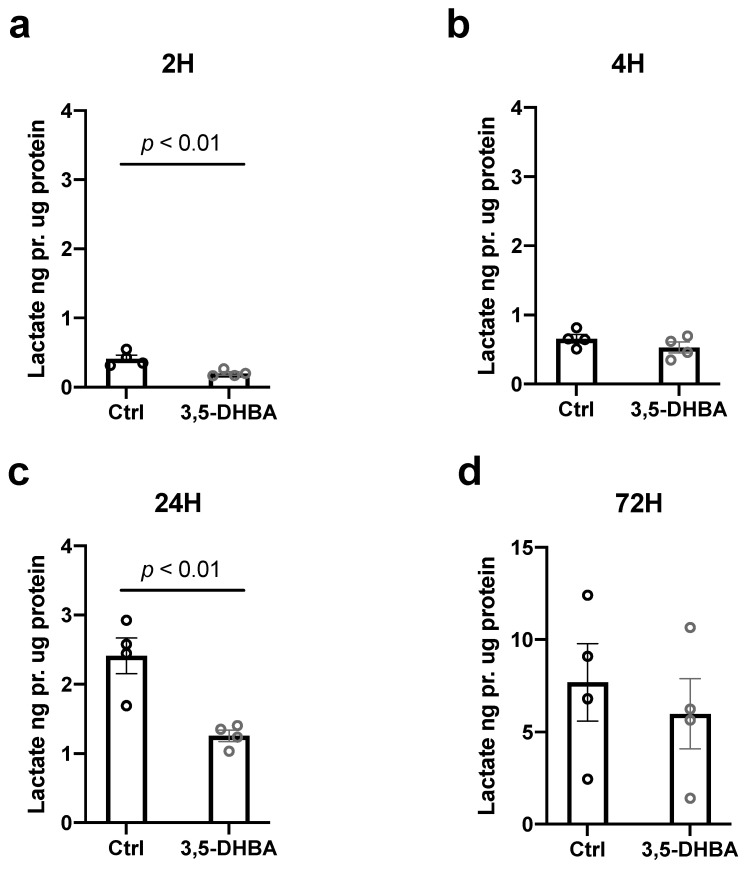
Extra-retinal lactate levels in retinal explants are decreased in response to 3,5-DHBA treatment. (**a**–**d**) The extra retinal concentrations of lactate measured in retinal explants incubated for 2, 4, 24, and 72 h. Lactate levels decrease during 2 and 24 h of incubation with 3,5-DHBA compared to controls. Values are normalized to mg of protein in each sample. Values are presented as mean ± S.E.M (*n* = 4 retinal quarters originating from four different whole retinas). Statistics: Unpaired *t*-test.

To further investigate metabolic implications of HCA_1_R activation, we measured extra retinal lactate levels after 2, 4, 24, and 72 h of treatment with 3,5-DHBA as an indirect measurement of glycolysis, the process by which glucose is converted to lactate. Extra-retinal lactate levels were significantly reduced during 2 h and 24 h of incubation with 3,5-DHBA (Figure 4a,c). These results could indicate that either glucose is metabolized and subsequent metabolites from glucose breakdown such as lactate is utilized intracellularly instead of being released to the surroundings during conditions of HCA_1_R activation or that glycolysis may be affected by HCA_1_R activation.

### 3.5. Glucose Metabolism Is Unaltered in Retinal Explants in Response to HCA_1_R Activation, Whereas Lactate Metabolism Is Slightly Altered

To further examine how glucose and/or lactate are metabolized intracellularly in the retina through the tricarboxylic (TCA) cycle in response to HCA_1_R activation, we measured ^13^C incorporation into TCA cycle metabolites (M+X) by GC-MS after incubation with either 6 mM [U-^13^C] glucose or 10 mM [U-^13^C] lactate for 2 h.

**Figure 5:**
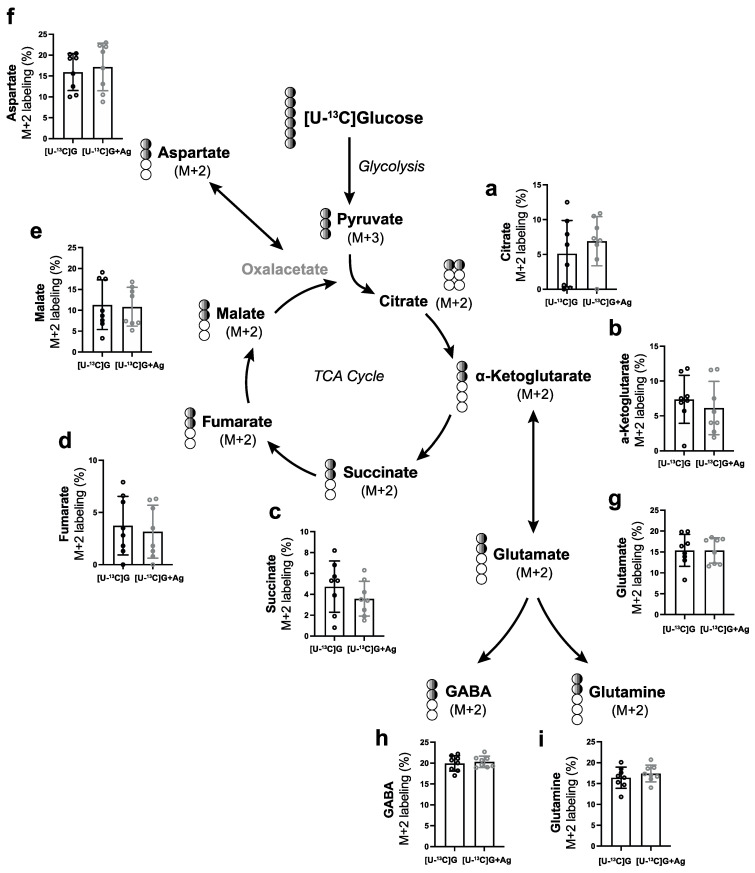
Retinal metabolism of [U-^13^C] glucose in response to 3,5-DHBA (Ag) treatment. (**a**–**i**) Retinal explants were incubated for 2 h with 6 mM [U-^13^C]glucose in the absence or presence of 5 mM of 3,5-DHBA. No significant changes in relative abundance of ^13^C labeling were found in any of the measured metabolites. Values are presented as mean ± S.D. (*n* = 8 retinal quarters originating from eight different whole retinas). Statistics: Unpaired *t*-test.

**Figure 6:**
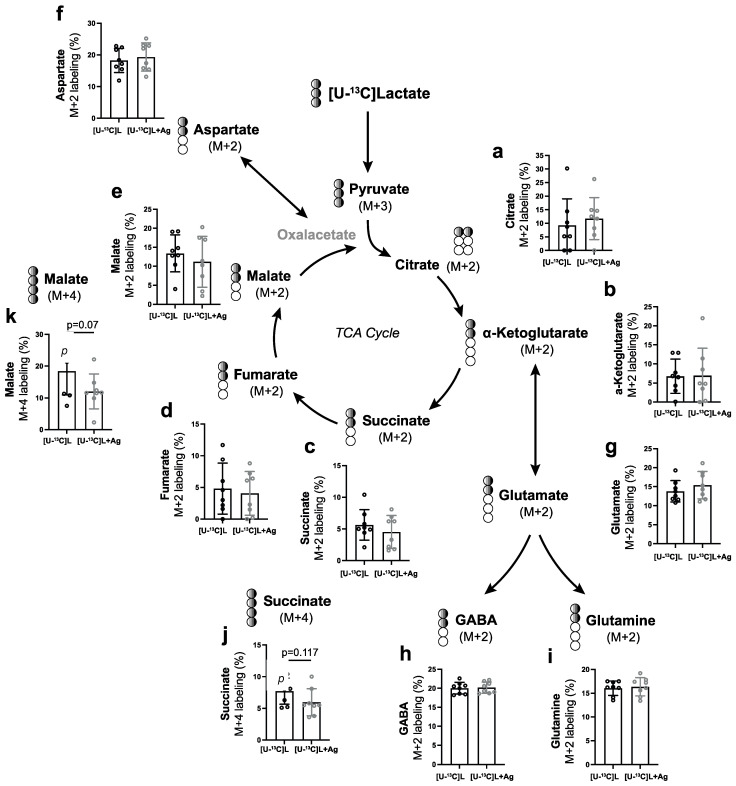
Retinal metabolism of [U-^13^C]lactate in response to 3,5-DHBA (Ag) treatment. (**a**–**k**) Retinal explants were incubated for 2 h with 10 mM [U-^13^C]lactate in the absence or presence of 5 mM of 3,5-DHBA. No significant changes in the relative abundance of ^13^C labeling were found in any of the measured metabolites. However, tendencies towards a decrease in malate (M+4) and succinate (M+4) were observed. Values are presented as mean ± S.D. (n = 8 retinal quarters originating from eight different whole retinas). Statistics: Unpaired *t*-test.

Both [U-^13^C] glucose and [U-^13^C] lactate are converted into pyruvate (M+3). Pyruvate (M+3) enters the mitochondria and is further metabolized to acetyl-coA (M+2), which undergoes metabolism in the TCA cycle. The relative abundances of [U-^13^C] glucose-derived citrate (M+2), alpha-ketoglutarate (M+2), glutamate (M+2), glutamine (M+2), GABA (M+2), succinate (M+2), fumarate (M+2), malate (M+2), and aspartate (M+2) remained unaltered in response to 3,5-DHBA (Ag) treatment (Figure 5). Similar findings seemed to be observed for the metabolism of [U-^13^C] lactate and subsequent incorporation of ^13^C into TCA metabolites (M+2) (Figure 6). However, we found differences in the relative abundance of succinate (M+4) (Figure 6j) and malate (M+4) (Figure 6k) when retinas were incubated with [U-^13^C] lactate and 3,5-DHBA (Ag), which did not reach statistical significance. This may indicate the presence of a truncated TCA cycle due to a high flux of intermediates in and out of the pathway, although this was not confirmed by an increase in aspartate (M+4).

### 3.6. Extra-Retinal Nitric Oxide Levels Are Unaltered in Response to HCA_1_R Activation

**Figure 7:**
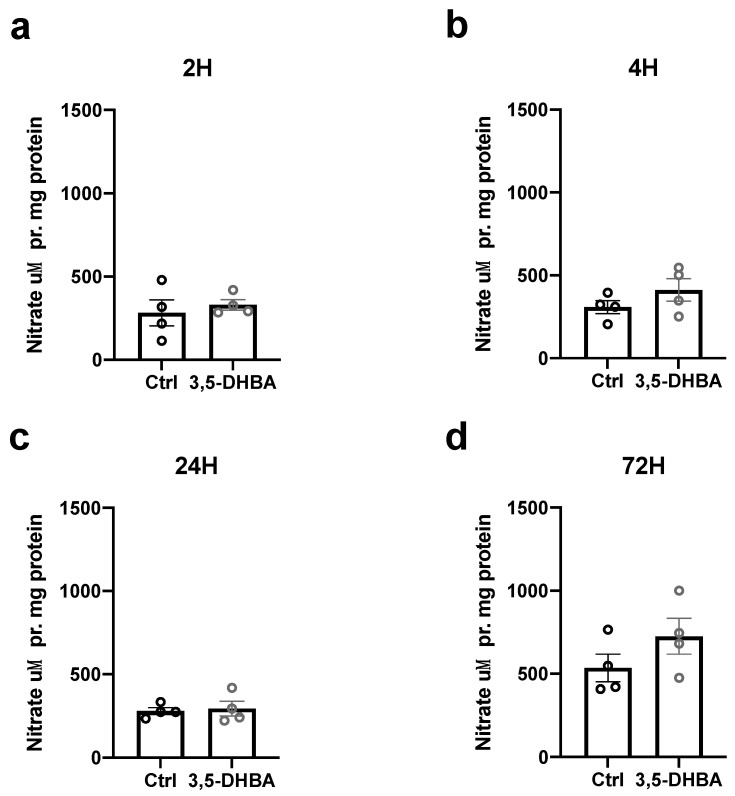
Nitric oxide concentration (μM) in response to 3,5-DHBA treatment. (**a**–**d**) Retinal explants were incubated for 2, 4, 24, and 72 h with 5 mM of 3,5-DHBA. No significant changes in extra retinal nitric oxide concentrations were found. Values are normalized to mg of protein in each sample. Values are presented as mean ± S.E.M (*n* = 4 retinal quarters originating from four different whole retinas). Statistics: Unpaired *t*-test.

HCA_1_R activation has been associated with anti-inflammatory responses [4,5] and pyruvate, which is stochiometrically related to lactate; the endogenous ligand of HCA_1_R has been shown to protect against oxidative and nitrosative stress [47,48,49]. Thus, we tested whether extraretinal concentrations of nitric oxide were correspondingly reduced in response to 3,5-DHBA treatment for 2, 4, 24, and 72 h. No significant changes in extraretinal nitric oxide levels were found (Figure 7).

## 4. Discussion

Emerging evidence points to a neuroprotective effect of lactate in models of traumatic brain injury [50] and ischemia [51]. However, there is limited knowledge about the beneficials effects of lactate in the retina. Recently, lactate treatment of RGCs was shown to improve cell viability and ATP production during stress induced by glucose deprivation [23]. In addition, systemic levels of lactate were shown to be lower in patients with well-defined degeneration of the RGCs, e.g., patients with normal tension glaucoma [52]. Thereby, suggesting that lactate may also serve as a neuroprotective molecule in the inner retina.

Besides being a crucial energy substrate in the retina [21,22,23], lactate has also been proposed to display regulatory functions upon activation of HCA_1_R [33,49,53]. Based on our recent study on HCA_1_R activation of cultured Müller glia [22], which showed improved glucose metabolism and mitochondrial function, we aimed to investigate neuroprotection and metabolic alterations in the entire retina in response to 3,5-DHBA, an HCA_1_R agonist-like treatment.

We found elevated retinal viability and RGC counts in the central subregions of the retina of C57/BL6J mice in response to 3,5-DHBA treatment as well as increased retinal viability during glutamate-induced cell death. RGC density is known to vary with a declining density along increasing radial distance [54]. Likewise, various animal models may result in different RGC death with a preference to either the peripheral or central retinal regions, with some glaucomatous models reporting sectorial RGC death corresponding to the area closest to the optic nerve head and disconnection of RGCs [55]. This may also explain why we only found 3,5-DHBA-induced neuroprotection in the central regions, since the optic nerve is chopped off to produce the retinal wholemount. The RGCs in the peripheral region may be assumed to thrive better compared to the RGCs of the damaged area in the center. The peripherally localized RGCs may therefore not be in need of the neuroprotection offered by HCA_1_R activation through 3,5-DHBA. The overall enhanced viability corresponded to a subsequent increase in ATP production, suggesting a metabolic boost with amplified metabolism. We also demonstrated that the release of lactate from the retina was decreased in response to HCA_1_R activation, possibly allowing internal conversion of glucose to pyruvate/lactate, which could be further processed in the TCA cycle in the mitochondria as acetyl-coenzyme-A. However, we were unable to verify this by GC-MS analysis of ^13^C labeling of metabolized [U^13^C]glucose nor [U^13^C]lactate in TCA cycle intermediates. We also did not observe any changes in levels of extra retinal nitric oxide.

HCA_1_R activates the Gi-mediated pathway by which adenylate cyclase is inhibited, resulting in a decrease of the secondary messenger cAMP [2,3], thereby altering various intracellular mechanisms associated with neuroprotection. Theoretically, a decrease in cAMP would lead to reduced protein kinase A, which usually facilitates the release of glutamate through an intracellular calcium increase [56]. Protein kinase A, as well as C, have been shown to mediate axon growth in the retina, whereas these effects were absent in retinas from HCA_1_R ^−/−^ transgenic mice [34]. Moreover, the HCA_1_R ligand lactate has been shown to increase Müller glia uptake of glutamate [21]. Combined, HCA_1_R activation would in principle lead to protection against glutamate excitotoxicity through reduced release and increased uptake. This has also been established in primary cortical neurons, where rapid calcium imaging revealed that HCA_1_R activation by either 3,5-DHBA or lactate reduced spontaneous calcium spiking by 40% in wild-type (WT) but not in HCA_1_R knock-out (KO) mice [7]. In the present study, we confirmed an improved retinal viability in response to 3,5-DHBA treatment during glutamate toxicity.

We also found an overall improvement in retinal viability in response to 3,5-DHBA agonist treatment. These findings are consistent with a previous study showing increased RGC growth in retinal explants from WT mice treated with lactate or 3,5-DHBA, which were not observed in HCA_1_R KO mice [34]. The neurogenesis effect of HCA_1_R has also been established in the cerebral ventricular and sub-ventricular zones of WT mice compared to HCA_1_R KO mice [12]. Other neuroprotective effects of HCA_1_R activation include regulation of inner retinal vasculature and increase of angiogenesis [57,58]. HCA_1_R KO mice have been shown to reduce inner retinal vessel formation [57]. Thus, a future study could investigate the correlation between intravitreal injections of 3,5-DHBA with RGC survival and investigate the impact on ocular blood flow and vascularity.

Metabolically, HCA_1_R activation is known to inhibit lipolysis of adipocytes in vitro and in vivo, suggesting that energy is preserved as fat during stress and short time starvation [28,35,59]. Linking these studies with HCA_1_R induced enhanced glucose metabolism and mitochondrial function in cancer cell lines and Müller glia [22,32,41,60]; HCA_1_R activation appears to instigate preferential metabolism of carbohydrates. In the present study, we observed lower extra retinal lactate amounts in the media when treated with the HCA_1_R like agonist, 3,5-DHBA. We assumed that this was due to enhanced metabolism of media-based glucose, as seen in Müller glia, thereby allowing lactate to be utilized internally or even bypassed as pyruvate entering the mitochondria for processing in the TCA cycle as AcetylcoA. However, this was not the case, since we did not find any changes in glucose metabolism (M+2, supplementary data (Figure S1) nor M+4) in response to 3,5-DHBA treatment measured by GC-MS. Certain cell types of the retina are known to enhance their glucose metabolism in response to HCA_1_R activation, such as Müller glia [22]; however, the metabolic effect of HCA_1_R activation may differ in other cell types of the retina. Since the present study is a measurement of the entire retina, the overall metabolic effect measured may be affected by other cells that are larger in numbers and do not show any metabolic change. Furthermore, we cannot exclude that lactate is secreted, yet bound to activate HCA_1_R, causing a subsequent decline in measurable lactate.

Nevertheless, we also observed lower amounts of labeled malate and succinate derived from [U^13^C] lactate metabolism when the retinas were incubated with 3,5-DHBA, although these differences did not reach statistical significance. This suggests the presence of a truncated TCA cycle, refilling the cycle downstream from the usual entry point to generate oxaloacetate. We measured labeled aspartate levels as an indicator for oxaloacetate. If a truncated TCA cycle is indeed taking place, aspartate should have been elevated. However, we observed no changes in the relative abundance of aspartate. This may be due to the fact that either labeled substrates were externally released from the retinal explants or internal unlabeled substrates were refilled in the TCA cycle, thereby causing a dilution of the labeling affected from HCA_1_R activation. Another possibility is the involvement of aspartate as substrate for aminotransferases, which could be relevant to measure expression levels of in future studies. A study by Rueda et al. confirmed the presence of truncated TCA cycle in murine retinas by establishing that certain retinal cells (e.g., Müller glia) could use TCA cycle to generate GTP and P-transferring kinases to produce ATP [61]. Lactate has previously been established as a preferred energy substrate in retinal cells [21,22,23], which could explain why the results obtained in the present study were not seen in the metabolism of [U^13^C] glucose. In addition, HCA_1_R expression has been shown to increase during glucose deprivation in Müller glia [22], possibly potentiating the effects of HCA_1_R activation on metabolism during glucose-free conditions. We have previously shown that the exposure to lactate increases the kinetic uptake of radiolabeled glutamate in human Müller glia (MIO-M1 cell line) as well as murine Müller glia [21]. Thus, glutamate may be one of the energy substrates entering the TCA cycle. This has also been confirmed by the incorporation of radioactive ^14^C labeling of glutamate into glutamine, aspartate, and lactate in rat Müller glia [62]. Nevertheless, we observed an increase in total ATP levels in response to HCA_1_R activation, which supports the idea of metabolism of other internal energy substrates. The results could also be an indication of elevated mitochondrial function as seen previously in cancer cells and retinal Müller cells [22,32,41]. Future assessments of retinal bioenergetics would therefore be of great relevance to clarify this.

Retinal degeneration has previously been associated with neuroinflammation [63], and neuronal cell death has previously been associated with the formation of nitrosative stress [64]. Thus, we aimed to investigate whether HCA_1_R activation subsequently protected against NO formation. Pyruvate, which is stoichiometrically linked to lactate (HCA_1_R ligand), has been shown to protect against NO formation in cultured retinal cells [48]. Yet, we did not observe any changes in extra retinal nitric oxide levels in the present study. Therefore, retinal neurodegeneration may be prevented by other anti-inflammatory means. In this regard, lactate-mediated HCA_1_R activation was shown to reduce various inflammatory markers, such as NLRP3 and interleukin (IL)-1β, in a mouse model of glaucoma [4]. Moreover, a down-regulation of pro-inflammatory markers, IL-1β,6,8; C-reactive protein; and matrix metalloproteinases has been shown to decrease in response to lactate treatment in WT mice compared with HCA_1_R KO mice [5]. Future studies should aim to elucidate similar anti-inflammatory responses in retinas treated with 3,5-DHBA.

The limitation of the present study is that no current antagonist for HCA_1_R exists, thus it is not possible to provide proof of mechanism (HCA_1_R activation) by reversing the reported effect, and unspecific action of 3,5-DHBA off-target cannot be ruled out.

Nevertheless, our study indicates that HCA_1_R activation is crucial for general retinal survival and RGC survival and may help to protect against glutamate-induced excitotoxicity. Thus, HCA_1_R appears to be a promising therapeutic target for alleviating retinal degenerative diseases. However, future research is needed to verify the neuroprotective effects of HCA_1_R activation in retinal tissue obtained from animal models and patients or surrogate measurements from humans diagnosed with inner retinal degenerative conditions.

## Data Availability

Not applicable.

## Supplementary Materials

The following are available online at https://www.mdpi.com/article/10.3390/cells11132098/s1, Figure S1: Retinal metabolism of [U-^13^C] glucose in response to 3,5-DHBA (Ag) treatment (M+4).
