# Supplementary figures and images for: Prevention of Cell Death by Activation of Hydroxycarboxylic Acid Receptor 1 (GPR81) in Retinal Explants
Source: Cells. 2022 Jul 2;11(13):2098. doi: 10.3390/cells11132098 (PMC9265426; doi:10.3390/cells11132098)

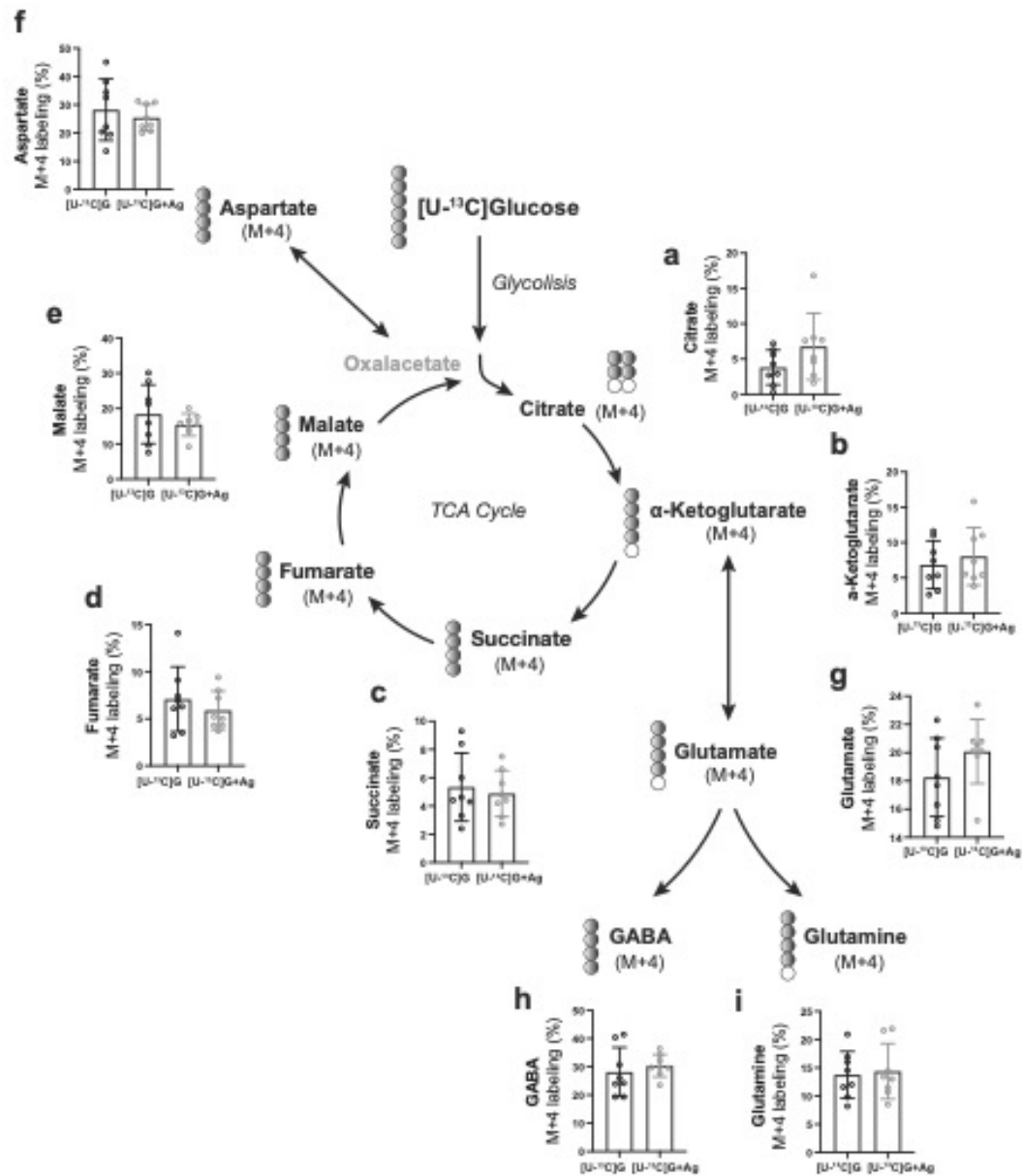

Figure S1: Retinal metabolism of [U-<sup>13</sup>C] glucose in response to 3,5-DHBA (Ag) treatment (M+4).

Supplement: Supplementary file 1 [file cells-11-02098-s001.zip › cells-1747144-supplementary.pdf]
